# Radiomics model based on contrast-enhanced computed tomography imaging for early recurrence monitoring after radical resection of AFP-negative hepatocellular carcinoma

**DOI:** 10.1186/s12885-024-12436-x

**Published:** 2024-06-07

**Authors:** Xuanzhi Yan, Yicheng Li, Wanying Qin, Jiayi Liao, Jiaxing Fan, Yujin Xie, Zewen Wang, Siming Li, Weijia Liao

**Affiliations:** 1 Laboratory of Hepatobiliary and Pancreatic Surgery, Affiliated Hospital of Guilin Medical University, No. 15, Lequn Road, Xiufeng District, Guilin, 541001 Guangxi P.R. China; 2 Department of Burns, Nanfang Hospital, Southern Medical University, Guangzhou, 510515 Guangdong P.R. China; 3 School of medical, Youjiang Medical University for Nationalities, Baise, 533000 Guangxi P.R. China; 4 Department of Hepatobiliary and Pancreatic Surgery, The Second Affiliated Hospital of Guilin Medical University, No. 212, Renmin Road, Lingui District, Guilin, 541100 Guangxi P.R. China

**Keywords:** Hepatocellular carcinoma, AFP-negative, Radiomics, Contrast-enhanced computed tomography, Recurrence prediction

## Abstract

**Background:**

Although radical surgical resection is the most effective treatment for hepatocellular carcinoma (HCC), the high rate of postoperative recurrence remains a major challenge, especially in patients with alpha-fetoprotein (AFP)-negative HCC who lack effective biomarkers for postoperative recurrence surveillance. Emerging radiomics can reveal subtle structural changes in tumors by analyzing preoperative contrast-enhanced computer tomography (CECT) imaging data and may provide new ways to predict early recurrence (recurrence within 2 years) in AFP-negative HCC. In this study, we propose to develop a radiomics model based on preoperative CECT to predict the risk of early recurrence after surgery in AFP-negative HCC.

**Patients and methods:**

Patients with AFP-negative HCC who underwent radical resection were included in this study. A computerized tool was used to extract radiomic features from the tumor region of interest (ROI), select the best radiographic features associated with patient’s postoperative recurrence, and use them to construct the radiomics score (RadScore), which was then combined with clinical and follow-up information to comprehensively evaluate the reliability of the model.

**Results:**

A total of 148 patients with AFP-negative HCC were enrolled in this study, and 1,977 radiographic features were extracted from CECT, 2 of which were the features most associated with recurrence in AFP-negative HCC. They had good predictive ability in both the training and validation cohorts, with an area under the ROC curve (AUC) of 0.709 and 0.764, respectively. Tumor number, microvascular invasion (MVI), AGPR and radiomic features were independent risk factors for early postoperative recurrence in patients with AFP-negative HCC. The AUCs of the integrated model in the training and validation cohorts were 0.793 and 0.791, respectively. The integrated model possessed the clinical value of predicting early postoperative recurrence in patients with AFP-negative HCC according to decision curve analysis, which allowed the classification of patients into subgroups of high-risk and low-risk for early recurrence.

**Conclusion:**

The nomogram constructed by combining clinical and imaging features has favorable performance in predicting the probability of early postoperative recurrence in AFP-negative HCC patients, which can help optimize the therapeutic decision-making and prognostic assessment of AFP-negative HCC patients.

**Supplementary Information:**

The online version contains supplementary material available at 10.1186/s12885-024-12436-x.

## Introduction

Primary liver cancer is the sixth most common malignant tumor in the world and the third leading cause of cancer-related deaths [1]. Hepatocellular carcinoma (HCC) is the main pathological type of primary liver cancer [2]. Surgical resection and liver transplantation are considered the main therapeutic options for early-stage HCC to prolong overall survival (OS) [3, 4]. However, because of the insidious onset of HCC and the lack of clinical symptoms in the early stages of the disease, the majority of HCC patients are already in advanced stages at the time of diagnosis [5]. In recent years, it has been reported that even if HCC patients received surgical resection, their prognosis was poor due to the metastatic and recurrent nature of HCC. Previous studies reported that the prognosis of patients with HCC, even after surgical resection, was not satisfactory, with a recurrence rate up to 70% at five years postoperatively [6]. In addition, there are reports that patients with HCC who suffer from recurrence in the early stages have a poorer prognosis than those who have a recurrence in the late stages of the disease [7]. Recurrences that occur within two years after surgery are considered early recurrences. Therefore, the development of systematic monitoring related to early diagnosis and early postoperative recurrence of HCC is urgently needed.

Serum alpha-fetoprotein (AFP) is now widely used as a biomarker for early diagnosis of HCC and assessment of postoperative recurrence. However, there are some limitations of AFP. First, AFP (threshold level of 20 ng/mL) has a high specificity (80–90%) but low sensitivity (40–60%) [6]. It has been reported that nearly 30% of HCC patients were AFP-negative, suggesting that routine serologic tests may overlook this group of AFP-negative HCC patients, thus affecting the accurate assessment of diagnosis and prognosis in AFP-negative patients. Secondly, AFP-negative HCC patients lack reliable and valid biological markers related to early postoperative recurrence. Therefore, the monitoring of postoperative recurrence in AFP-negative HCC patients needs to be further strengthened and improved. In recent years, researchers found that C-reactive protein may be a biomarker for AFP-negative HBV-associated HCC [8]. In the search for biomarkers associated with AFP-negative HCC, some studies have shown that a nomogram constructed by multiple indicators can be used to assist in the assessment of prognostic status after surgery for AFP-negative HCC [9]. Currently, some studies have suggested that the inflammation-associated complex markers GLR [gamma-glutamyl transpeptidase (U/L) / lymphocyte (10^9^/L)] and NrLR [neutrophil (10^9^/L) × gamma-glutamyl transpeptidase (U/L) / lymphocyte (10^9^/L)] may be associated with the prognosis of patients with AFP-negative HCC after radical surgery [10, 11], which further demonstrated that multiparameter marker may be a potential marker for early postoperative recurrence in AFP-negative HCC. AGPR is an emerging composite marker in recent years with the formula [alkaline phosphatase (U/L) + gamma-glutamyl transpeptidase (U/L)]/platelet (10^9^/L) (AGPR). AGPR has been reported to be useful in the assessment of liver fibrosis and cirrhosis [12]. However, the association of AGPR with HCC remains unclear.

Radiomics, as an emerging medical image analysis method in recent years, performs high-throughput extraction of quantitative metric features through a series of data mining algorithms or statistical analysis tools [13, 14] and uses them to predict the patient’s risk of morbidity and prognosis, and to obtain prognostic and predictive information that is useful for clinical decision support.

With the rapid development of medical imaging, researchers are coming to realize that imaging features in or around tumors may be associated with a range of clinical prognoses and underlying genetic patterns across a range of tumor types [15]. Current medical imaging consists of ultrasound (US), computed tomography (CT), and magnetic resonance imaging (MRI). All of these medical images play a vital role in the screening and diagnosis of HCC, but there are limitations in conventional imaging compared to radiomics. Conventional imaging focuses only on the apparent image visible to the naked eye, mainly the size and relative anatomical location of the tumor, but ignores other important information about the tumor (e.g., smoothness of tumor margins, apparent diffusion, envelope clarity, etc.). Moreover, conventional imaging is highly dependent on the experience and judgment of radiologists, who may inevitably miss or have difficulty in quantifying some key details. Emerging radiomics allows for a more comprehensive evaluation of tumors under non-invasive conditions [16]. In recent years, reports have suggested that radiomic features may be useful diagnostic and prognostic biomarkers for HCC and other tumor types. It has also been reported that medical imaging was capable of detecting tumors earlier, but its lower sensitivity to early-stage tumors limits the efficiency of a single image to diagnose tumors [17]. Therefore, combining clinical and serological markers with imaging signatures may improve this. Survival and prognostic status of patients with AFP-negative HCC can be somewhat improved if their status can be assessed preoperatively. A recent study has shown that obtaining relevant features based on preoperative imaging of HCC patients can be used to predict early recurrence after HCC surgery [18], further demonstrating that radiomic features based on preoperative contrast-enhanced computed tomography (CECT) can have the potential to predict the prognosis of AFP-negative HCC after surgery.

Due to the lack of reliable assessment of early postoperative recurrence in patients with AFP-negative HCC, there is an urgent need to develop an accurate and scientific tool to predict early postoperative recurrence in AFP-negative HCC. The aim of this study was to develop a radiomic model based on radiomic features and clinical variables to predict early recurrence in patients with AFP-negative HCC undergoing radical resection, evaluate the performance of the model, and validate its feasibility in clinical applications.

## Materials and methods

### Patient selection and data collection

This study was approved by the Research Ethics Committee of the Affiliated Hospital of Guilin Medical University in strict adherence to the principles of the Declaration of Helsinki, and informed consent was obtained from all subjects.

**Fig. 1:**
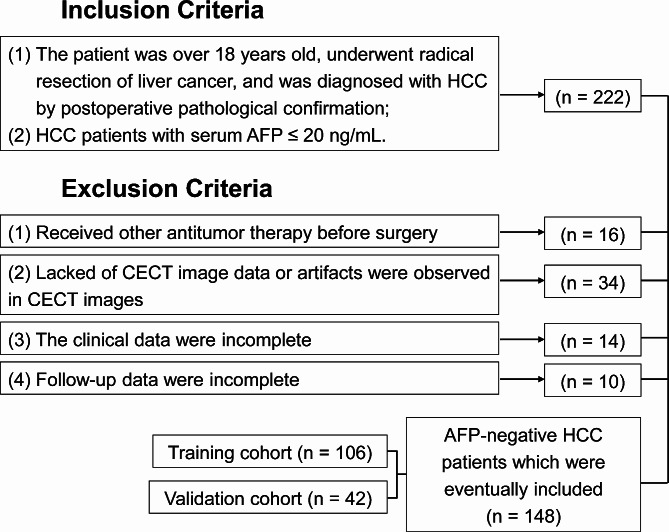
Flowchart of the patient selection process. HCC: Hepatocellular carcinoma; CECT: Contrast-enhanced computed tomography; AFP: Alpha fetoprotein

We regressively included 148 patients with AFP-negative HCC who underwent radical surgery from January 2014 to December 2020 at the affiliated hospital of Guilin Medical University and were confirmed by pathology. They fulfilled the inclusion and exclusion criteria (Fig. 1).

The inclusion criteria for this study were as follows: (1) patients aged 18 years or older who were treated with radical resection for hepatocellular carcinoma and were diagnosed with HCC on postoperative pathological conformation, and (2) HCC patients with serum AFP ≤ 20 ng/mL. The exclusion criteria were as follows: (1) previous antitumor therapy; (2) the presence of other primary malignancies; (3) incomplete or unavailable clinical data; (4) lack of CECT image data or artifacts observed in CECT images; and (5) immune and hematologic diseases or active infections.

HCC was diagnosed on the basis of clinical characteristics, radiological examination, hepatic arteriography, AFP level, and postoperative pathological examination, with reference to the “Clinical Diagnosis and Staging of Primary Hepatocellular Carcinoma” formulated by the Hepatocellular Carcinoma Specialized Committee of the Chinese Anti-Cancer Association. Radical resection was defined as a complete resection of the tumor mass based on two observations not less than 4 weeks interval, with pathological confirmation of the negative margins and no residual tumor or new lesions [19]. All tumor tissue samples were independently diagnosed by at least two experienced pathologists. All patients underwent CECT scanning and hematological examination prior to surgery.

### Follow up

Postoperative outpatient review is the main form of follow-up, beginning at one month postoperatively. It is repeated every two months for two years after surgery and every six months thereafter. Among them, the routine postoperative review includes physical examination, blood routine, liver function, kidney function, AFP level, and abdominal ultrasound. If the examination results are abnormal or tumor recurrence is suspected, a CECT scan is recommended. And for patients who did not attend the return visit, the follow-up information was obtained by telephone. Overall survival (OS) was defined as the time from surgery to death or the last follow-up, and recurrence-free survival (RFS) was defined as the time from surgery to the first intra- and/or extrahepatic recurrence of the tumor, death, or the last follow-up.

### Radiomics signature acquisition

**Fig. 2:**
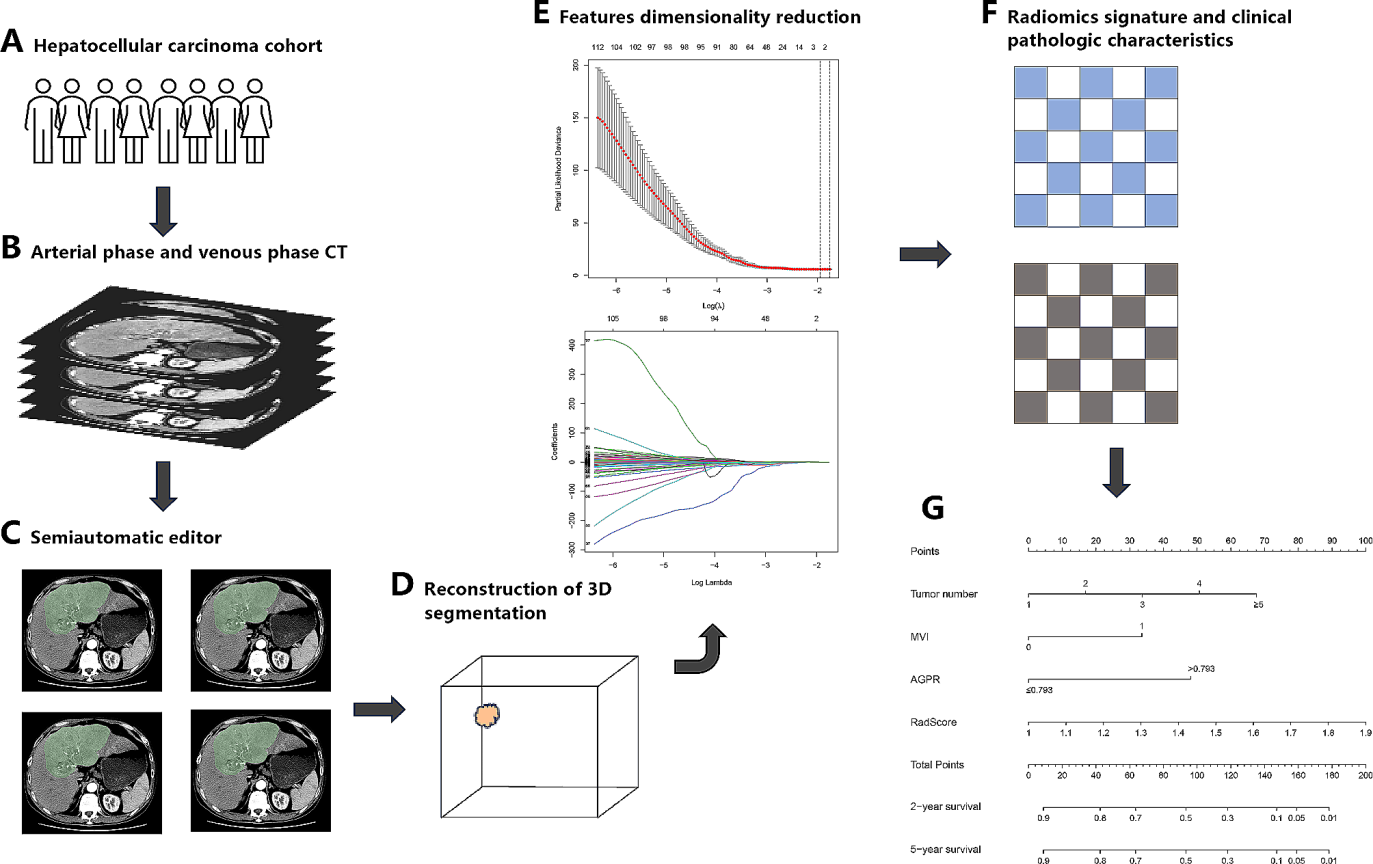
Workflow of model construction and validation. (**A**) Screening of AFP-negative HCC patients. (**B**) CECT image acquisition and preprocessing. (**C**, **D**) Semiautomated segmentation of ROI and reconstruction of 3D segmentation. (**E**, **F**) Radiomic features associated with early recurrence were extracted from ROIs, and the best features were selected by 10-fold cross-validation using LASSO. The clinical features associated with early recurrence were then extracted using a machine learning algorithm. (**G**) A nomogram was constructed by combining radiomic features and clinical features. AFP: Alpha fetoprotein; HCC: Hepatocellular carcinoma; CECT: contrast-enhanced computed tomography; ROI: region of interest

We use computer-aided methods to extract quantitative features from images. These features include morphological, textural, and dynamic enhancement features. The following are several manual steps for obtaining radiomic features associated with AFP-negative HCC: (1) image acquisition and preprocessing; (2) region of interest (ROI) selection; (3) extraction of radiomic features from the ROI; and (4) feature downscaling and construction of radiomics Score (RadScore) (Fig. 2A-G). Detailed procedures can be found in previous related work [20, 21].

### Image acquisition and pre-processing

All CECT images in this study conformed to the criteria set forth in the American Guidelines for the Study of Liver Diseases [17]. CECT images were exported in Digital Imaging and Communications in Medicine (DICOM) format from the Image Archiving and Communication System database. All DICOM images were converted to the Neuroimaging Informatics Technology Initiative (NITI) format by the SimpleITK package (version 1.2.0) of PYTHON software (version 3.7).

The acquisition and preprocessing of CECT images for each patient was performed according to AASLD guidelines. Abdominal CECT scans were performed using two scanners: a Lightspeed VCT XT (GE Healthcare, USA) and an Optima CT660 (GE Healthcare, USA). The scanners were operated in cine mode with the following parameters: tube voltage 120 kV, automatic tube current modulation (MA), noise index 8, tube speed 600 ms, pitch 0.985:1, and collimator 0.625 mm. Arterial-phase (AP) imaging was performed using the contrast tracking technique. Lopromide (Ultravist 300, Bayer-Schering Pharma, Germany) was administered intravenously via the anterior vein at a rate of 4 ml/s in a volume of 1.5 mL/kg. The arterial and portal phase slices were 5 mm thick and spaced at 5 mm intervals. All images were reconstructed to be 1.25 mm thick and 1.25 mm spaced. All data were transferred to an advanced workstation (AW4.7). And then the images in AP and Venous-phase (VP) were normalized using z-Score normalization to obtain a standard normal distribution of image intensities and resampled to 1 × 1 × 1 mm^3^ voxels by PYTHON (V4.2, https://www.python.org) and the open-source SIMPLE-ITK package) to create comparable and reproducible radiomics analysis.

### Region of interest (ROI) selection

Tumor segmentation was performed using 3D Slicer software (version 4.11.20210226). ROIs were drawn on the horizontal plane from the upper to the lower border of each layer of the tumor. Images were independently reviewed by two blinded radiologists with 7 and 8 years of experience, and a third radiologist resolved any discrepancies. For patients with multiple tumors, only the largest tumor was selected.

### Radiomic features extraction

CECT image normalization and radiomic feature extraction were performed using the Python software Pyradiomics package (version 3.0.3). Radiomic features extracted from ROI included first-order features, shape features (2D and 3D), gray-level covariance matrix features, gray-level size band matrix features, gray-level stroke length matrix features, gray-level dependency matrix features, and adjacent gray-level hue difference matrix features.

### Feature downscaling and construction of radiomic features

The radiomic feature values were normalized using z-Score (z = (x-µ)/σ) from the training cohort. And a 10-fold cross-validation method using the least absolute shrinkage and selection operator (LASSO) in the training cohort was used to reduce the dimensionality of the data. The best radiomic parameters are selected and applied to the validation cohort.

### Construction of prediction model and performance evaluation

Three models for predicting recurrence status in AFP-negative HCC were developed: the radiomics model (RM), the clinical model (CM), and the clinical-radiomics model (CRM). CM lacked radiomic features, RM relied solely on radiomic features, and CRM included clinical and radiomic features.

### Data analysis and statistical processing

All statistical analyses were performed using R software (version 4.11, https://www.r-project.org/).The Kolmogorov-Smirnov test tested whether continuous variables were normally distributed. Normally and non-normally distributed variables were expressed as mean ± standard deviation (std) or median with interquartile range (IQR), respectively. Student’s t-test or Mann-Whitney u-test was used for continuous variables, and the Pearson χ2 test or Fisher exact probability test was used for categorical variables. The threshold for statistically significant differences was set at *p* < 0.05. One-way and multifactor regression analyses were performed on the training cohort using Cox proportional risk models to identify independent predictors for nomogram construction. RMS and regplot packages were used to build the nomograms and calibration curves. ROC curve analysis was performed using the timeROC package.

## Results

### Demographic and clinicopathologic characteristics

A total of 148 AFP-negative HCC patients (males, *n* = 135; females, *n* = 13) were included in this study according to the inclusion and exclusion criteria. The mean age of the patients was 55.01 years old (range 30–84 years old). Patients were randomized in this study into a training cohort (*n* = 106) and a validation cohort (*n* = 42). Demographic and clinicopathologic characteristics are summarized in Table 1. Statistically verified variables did not differ significantly between the two cohorts.

**Table 1:** The demographic and clinical-pathologic characteristics of patients

Variable	Training cohort (*n* = 106)	Validation cohort (*n* = 42)	*p*-value
Age, years (mean ± std)	56.07 ± 10.60	52.36 ± 12.04	0.101
Gender (male/female)	96/10	39/3	0.643
Tumor number (mean ± std)	1.32 ± 0.72	1.26 ± 0.80	0.679
Tumor size, cm (mean ± std)	6.42 ± 4.21	6.32 ± 3.45	0.883
MVI (absent/present)	67/39	32/10	0.115
BCLC (0 + A/B-C)	74/34	32/10	0.308
WBC, ×10^9^/L (mean ± std)	6.59 ± 2.80	6.11 ± 1.77	0.178
PLT, ×10^9^/L (mean, IQR)	201.19 (143.25–234.50)	168.98 (131.5-219.75)	0.157
LYMPH, ×10^9^/L (mean ± std)	1.74 ± 0.65	1.83 ± 0.66	0.447
NEUT, ×10^9^/L (mean ± std)	3.98 ± 2.09	3.53 ± 1.45	0.143
TB, µmol/L (mean ± std)	13.66 ± 8.22	14.31 ± 5.01	0.555
DB, µmol/L (mean ± std)	5.51 ± 3.11	5.43 ± 2.34	0.864
ALB, g/L (mean ± std)	39.06 ± 5.27	39.77 ± 3.89	0.370
PA, mg/L (mean, IQR)	225.46 (148.40-226.99)	197.76 (167.29-240.66)	0.331
ALP, U/L (mean, IQR)	122.36 (69.00-97.75)	77.65 (63.22–85.36)	0.056
GGT, U/L (mean, IQR)	98.43 (35.25-123.04)	75.41 (32.44–85.70)	0.191
AGPR (≤ 0.793/> 0.793)	48/58	20/22	0.800
ALT, U/L (mean, IQR)	35.39 (18.46–41.09)	40.29 (20.90–45.50)	0.445
AST, U/L (mean, IQR)	40.94 (23.15–41.75)	39.15 (22.83–38.71)	0.832
HBsAg (negative/positive)	30/76	9/33	0.379

MVI, microvascular invasion; BCLC, barcelona clinic liver cancer; WBC, white blood cell; PLT, platelet; LYMPH, lymphocyte; NEUT, neutrophil; TB, total bilirubin; DB, direct bilirubin; ALB, albumin; PA, prealbumin; ALP, alkaline phosphatase; GGT, gamma-glutamyl transpeptidase; AGPR, [alkaline phosphatase (U/L) + gamma-glutamyl transpeptidase (U/L)]/platelet (10^9^/L); ALT, alanine aminotransferase; AST, aspartate aminotransferase; HBsAg, hepatitis B surface antigen; std, standard deviation, IQR, interquartile range

### Radiomics characteristics and the basis of the radiomics signature

**Fig. 3:**
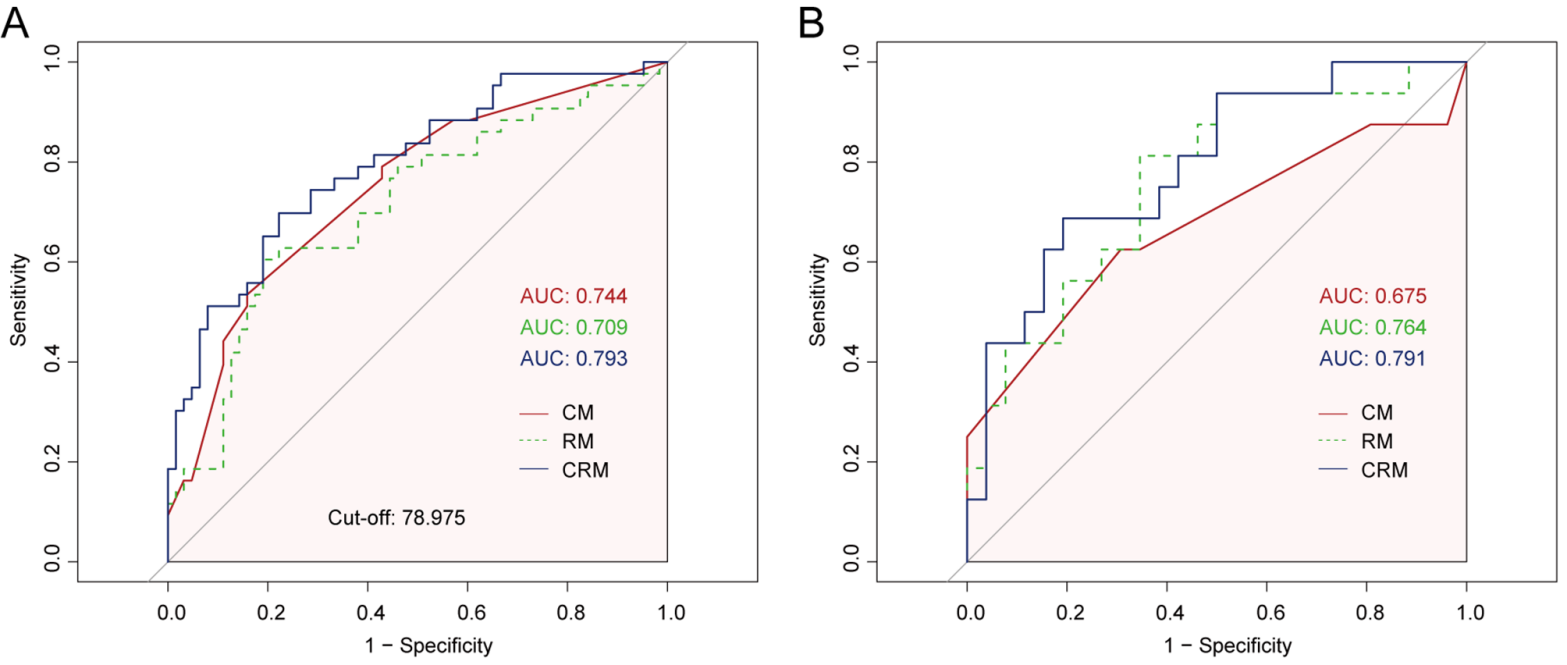
Evaluation of three models to predict early recurrence. (**A**) ROC curves for different models predicting recurrence in the training cohort. (**B**) ROC curves for different models predicting recurrence in the validation cohort. RM: Radiomics Model; CM: Clinical Model; CRM: Clinical-Radiomics Model

A total of 1977 radiomic features were extracted from the ROIs in this study. Based on the training cohort, we used the LASSO algorithm to reduce the radiomic features to two disease-free survival (RFS)-related features. A radiomics score (RadScore) was calculated for each HCC patient based on the identified features and their respective coefficients. The radiomics model (RM) constructed on the basis of the radiomics score showed good early recurrence prediction ability. The AUCs of RM were 0.709 and 0.764 in the training and validation groups, respectively (Fig. 3A and B). The radiomic signature formula and the results of the selected features are shown in the Supplementary File.

### Nomogram development and validation

**Fig. 4:**
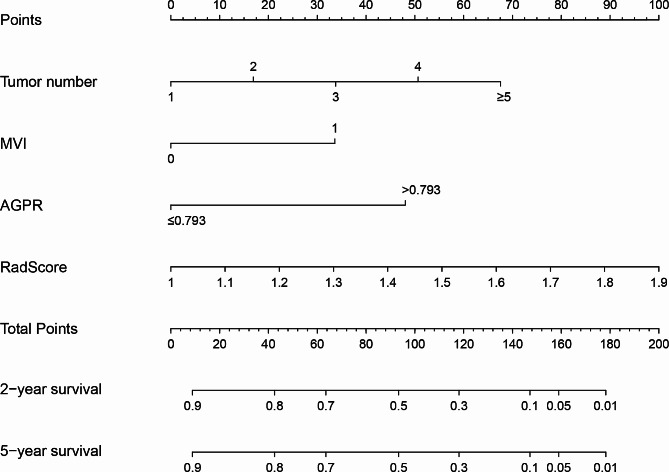
A nomogram was constructed based on CECT features and clinical characteristics associated with early recurrence. MVI: microvascular invasion; AGPR: [alkaline phosphatase (U/L) + gamma-glutamyl transpeptidase (U/L)]/platelet (10^9^/L)

**Fig. 5:**
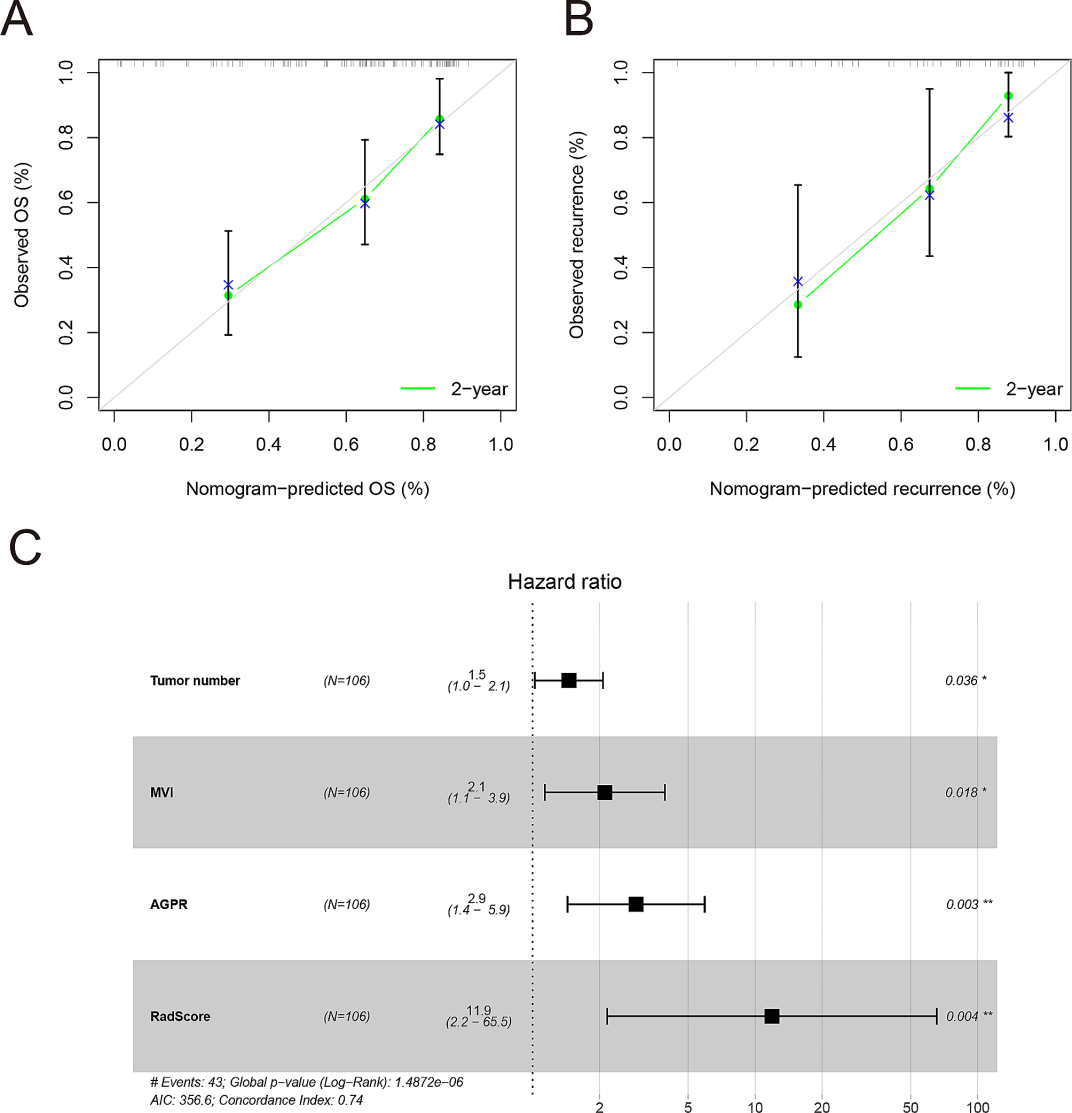
The convergence curves of predicted outcomes and forest plots of variables in the training cohort were subjected to multiple regression analysis. (**A**, **B**) Calibration curves of the nomogram in the training cohort (**A**) and validation cohort (**B**). In the calibration curve, the nomogram predicted survival probability is represented on the x-axis, and the actual outcome of RFS is represented on the y-axis. A closer fit of the colored lines to the ideal gray line indicates better accuracy in predicting RFS. (**C**) Forest plot of variables in multivariate regression analysis in the training cohort. Variables with a P value less than 0.05 were considered to be significantly associated with RFS in patients with AFP-negative hepatocellular carcinoma. RFS: recurrence-free survival

In this study, on the basis of univariate cox regression analysis (Table 2), four variables with *p* < 0.05 were selected for multivariate regression analysis. The results of the multivariate regression analysis were displayed as a forest plot (Fig. 5C). Combined with these results, it is clear that tumor number, microvascular invasion (MVI), AGPR (threshold of 0.793), and RadScore were identified as independent predictors of RFS. The above four variables were incorporated into the clinic-radiomics prediction model (Fig. 3A and B) and visualized by a nomogram (Fig. 4). The AUCs of the clinic-radiomics model (CRM) in the training cohort and validation cohort were 0.793 [accuracy: 0.755; C-index: 0.781; 95% CI: 0.749–0.905] and 0.791 [accuracy: 0.762; C-index: 0.735; 95% CI: 0.650–0.932], respectively (Table 3). In contrast, the clinical model (CM) constructed from tumor number, MVI and AGPR (threshold of 0.793) had AUCs of 0.744 and 0.675 in the training cohort and validation cohort, respectively. The calibration curve showed good agreement between the predicted survival probability and the actual survival probability (Fig. 5A and B).

**Table 2:** Univariate and Multivariate Cox regression analysis in the training cohort

Variables	Univariate Cox regression analysis				Multivariate Cox regression analysis		
	HR	95%CI	*p*-value		HR	95%CI	*p*-value
Age, years	0.992	0.965–1.020	0.563				
Gender (male vs. female)	2.311	0.559–9.559	0.247				
Tumor number	1.504	1.125–2.010	**0.006**		1.457	1.025–2.072	**0.036**
Tumor size, cm	1.034	0.976–1.095	0.258				
MVI (present vs. absent)	2.499	1.371–4.557	**0.003**		2.116	1.137–3.936	**0.018**
TB, µmol/L (> 21 vs. ≤ 21)	0.972	0.922–1.026	0.314				
AGPR (> 0.793 vs. ≤ 0.793)	3.179	1.599–6.320	**0.001**		2.92	1.437–5.936	**0.003**
HBsAg (positive vs. negative)	2.032	0.942–4.384	0.071				
Radiomics score	27.371	5.131-146.013	**< 0.0001**		11.905	2.165–65.470	**0.004**

MVI, microvascular invasion; TB, total bilirubin; AGPR, [alkaline phosphatase (U/L) + gamma-glutamyl transpeptidase (U/L)]/platelet (10^9^/L); HBsAg, hepatitis B surface antigen; HR, hazard ratio; CI, confidence interval

**Table 3:** Parameters related to early recurrence prediction model for AFP-negative HCC

Groups	Model	Accuracy (%)	AUC	C-index	95%CI
Training cohort	CM	0.604	0.744	0.747	0.650–0.839
	RM	0.642	0.709	0.655	0.606–0.812
	CRM	0.755	0.793	0.781	0.749–0.905
Validation cohort	CM	0.667	0.675	0.641	0.497–0.854
	RM	0.714	0.764	0.722	0.614–0.915
	CRM	0.762	0.791	0.735	0.650–0.932

CM, clinical model; RM, radiomics model; CRM, clinical-radiomics model; AUC, area under curve; CI, confidence interval

### Evaluation of clinical utility and risk stratification ability

**Fig. 6:**
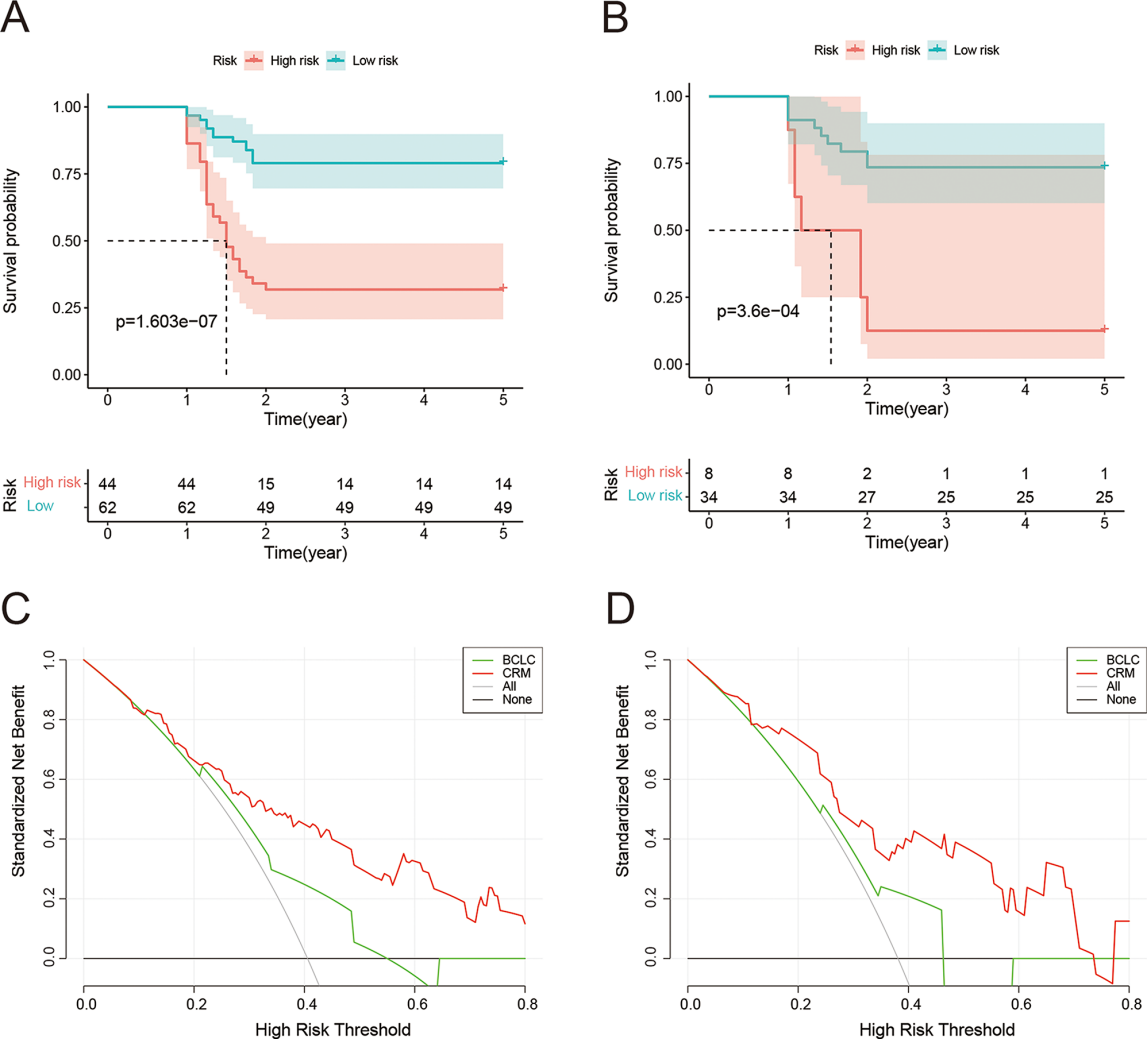
A depiction of the survival risk stratification curve and decision curve. (**A**, **B**) Kaplan-Meier curves between the low- and high-risk subgroups of overall survival in the training cohort (**A**) and validation cohort (**B**). (**C**, **D**) Decision curve analysis of the nomogram and Barcelona Clinic Liver Cancer staging system model in predicting RFS for the training cohort (**C**) and validation cohort (**D**)

The results of the DCA curves showed that the net benefit of CRM in predicting 2-year RFS at a reasonable threshold probability was higher than that of the BCLC staging system model (Fig. 6C and D). To further explore the nomogram risk stratification ability, we calculated the total nomogram score for each patient. In both the training and validation cohorts, the total score conformed to a normal distribution. We first obtained the optimal cutoff value of the nomogram through the ROC curve. And then divided patients into the low-risk (≤ 78.975) and high-risk (> 78.975) groups, respectively. Kaplan-Meier curves showed that patients in the low-risk subgroups had a significantly longer RFS, with *P* < 0.005 in both cohorts (Fig. 6A and B).

## Discussion

Due to the lack of early recurrence indicators in AFP-negative HCC patients, we comprehensively assessed the radiomics score (RadScore) based on the CECT of 148 AFP-negative HCC patients at Guilin Medical College Hospital, which was closely associated with early recurrence of AFP-negative HCC. And then, we also incorporated some serological indicators and clinical characteristics that may be associated with early recurrence with the RadScore and established a nomogram for predicting RFS after radical hepatectomy in patients with AFP-negative HCC. Our study supports the use of CECT-based radiomics in the monitoring of early recurrence after AFP-negative HCC surgery. Previous studies have also explained the potential of radiomics in predicting HCC recurrence [22–24]. However, few studies have focused on early recurrence after resection of AFP-negative HCC. In addition, the model constructed in this study categorized patients with AFP-negative HCC into two subgroups with high and low risk of early recurrence, and the difference in RFS between the two subgroups was significant.

With the advancement of medical technology tools, it has been gradually realized that medical images contain many subtle features that are potentially more significant for tumor treatment and diagnosis [16]. Although the histopathologic features of HCC can largely determine the prognosis of patients, this is highly dependent on invasive biopsy or postoperative testing. This means that obtaining histopathologic features related to HCC is not only potentially risky but also potentially problematic, such as not being able to obtain them in a timely manner. Medical imaging, on the other hand, provides a more complete view of the tumor and gives information about biology and heterogeneity. Intratumor heterogeneity has been reported to be common in a wide range of tumors and is strongly correlated with clinical outcomes [25]. Radiologists often find it difficult to analyze these details from conventional imaging pictures, and radiomics overcomes these limitations of conventional radiology to a certain extent, as radiomics is capable of extracting quantitative high-throughput imaging features from medical images and computer-assisted data mining, which greatly improves the use of image information. In addition, it is now generally accepted that radiomics is also possible to identify whether a tumor is recurrent or primary to some extent by detecting the corresponding gene profile and tumor microenvironment [26, 27]. Subsequently, Villanueva et al. developed a composite prognostic model for HCC recurrence based on gene expression in tumor tissue [28]. However, most cases of recurrent HCC occur within 2 years after surgery, which is considered to be true early recurrence. Numerous studies have shown that postoperative patients with early recurrence of HCC have worser outcomes than those with late recurrence [29, 30]. Therefore, monitoring early recurrence is of great importance in the postoperative management of HCC, and even more significant for patients with AFP-negative HCC who lack a reliable means of assessing postoperative recurrence. In recent years, a large number of reports have claimed that the immune microenvironment plays a crucial role in the early recurrence of HCC. Early recurrent HCC at single-cell resolution has been reported to possess unique features [31]. In addition, changes in various metabolic microenvironments, such as lipogenesis and glycolysis, may also induce recurrence [32, 33]. The results of the current studies undoubtedly illustrate the complexity of recurrent tumor and the lack of reliable and effective means of identifying recurrent HCC. Although many staging systems have been applied to the diagnosis and treatment of HCC, including the AJCC TNM staging system as well as the BCLC and Child-Pugh staging systems [34, 35], however, none of them can predict recurrence. Serum AFP level has long been used as a predictor of recurrence after hepatectomy and liver transplantation [36, 37], but it may be an ineffective tool for recurrence assessment in AFP-negative HCC patients. Therefore, the development of postoperative recurrence assessment tools for AFP-negative HCC patients is urgently needed.

In recent years, preoperative imaging has played an important role in the effective diagnosis and treatment of HCC, of which CECT is one important component. Hepatic resection is considered suitable for HCC patients with BCLC grades A and B and is the main treatment strategy at this stage. In consideration of the extremely high rate of recurrence after resection and the presence of AFP-negativity in roughly 1/3 of HCC patients, we decided to use radiomics to explore more digital features from CECT images that are associated with early postoperative recurrence in AFP-negative patients. Previous studies have predicted early postoperative recurrence for HCC by imaging features [23, 38]. And in this study, we determined the radiomics score based on two relevant radiomic features (wavelet.LLH_firstorder_maximum and wavelet.LHL_glszm_ZoneEntropy.1). Wavelet.LLH_firstorder_maximum and wavelet.LHL_glszm_ZoneEntropy.1 represent the first-order statistical characteristics of the tumor region: maximum gray intensity and zone entropy of the gray size zone matrix, respectively. We propose the hypothesis that the characteristics implied behind the two features may explain malignant behavior in the early recurrence of HCC. Although the mechanism by which these two key imaging features influence tumor biology is not clear, studies have indicated that first-order statistical features and GLSZM features are associated with tumor prognosis and recurrence [15]. In addition, radiomics has its own advantages, such as it can convert image information into digital information, predict MVI for HCC [15], reconstruct HCC gene expression profiles, and obtain tumor-associated candidate genes or proteins, etc [39, 40]. Thus, deep mining of the biological information contained in medical imagings can help to elucidate the molecular mechanisms of hepatocarcinogenesis. Therefore, we combined radiomic features and serological characteristics to assess the risk of early postoperative recurrence in AFP-negative HCC, which was confirmed in an independent validation cohort. Our findings suggest that non-invasive features have favorable value for early recurrence surveillance for AFP-negative HCC and may guide clinical decision-making.

The current study has several limitations. Firstly, this study is a single-center retrospective study. On one hand, limited study data may be the main limitation of a single-center study, and on the other hand, inherent bias in retrospective analyses is unavoidable. Secondly, even if radiomic features are extracted by a standard process, these features may be different and heavily dependent on the CT machines and medical centers. Thirdly, with the development of multimodal radiomics, Gd-EOB-DTPA-enhanced MR or 18 F-FDG PET/CT have been widely used, and several studies have shown that these means have higher response rates, especially in adipose tissues and tumor capsules [41, 42]. Therefore, we should perform prospective investigations using multimodal radiomics to confirm our results.

## Conclusion

This study demonstrates the potential of a radiomic approach in the surveillance of recurrence in AFP-negative HCC. Prediction model combining radiomic features, tumor number, MVI and AGPR may be useful for postoperative prognosis prediction and clinical treatment decision-making in patients with AFP-negative HCC. Further studies and clinical validation will help promote the clinical application of this model. We hope to further optimize and standardize this imaging model to enhance the application of radiomics in tumor.

### Electronic supplementary material

Below is the link to the electronic supplementary material.

Supplementary Material 1

## Data Availability

The data presented in this study are available on request from the corresponding author.
